# The school-life balance effect on acquiring cross-disciplinary competences in VET: disruption or continuity during COVID-19?

**DOI:** 10.1007/s12186-023-09314-1

**Published:** 2023-03-13

**Authors:** Angelina Sánchez-Martí, Anna Ciraso-Calí, Héctor Fernández-Sequi, Pilar Pineda-Herrero

**Affiliations:** 1 Department of Applied Pedagogy, Serra Húnter Fellow, Universitat Autònoma de Barcelona, Barcelona, Spain; 2 Department of Applied Pedagogy, Universitat Autònoma de Barcelona, Barcelona, Spain; 3 Secondary Education Teacher of the Department of Education, Generalitat de Catalunya, Barcelona, Spain; 4 Department of Theories of Education and Social Pedagogy, Universitat Autònoma de Barcelona, Barcelona, Spain

**Keywords:** School-life balance, VET, Competences, COVID-19, Non-traditional students

## Abstract

Considering that teleworking and online training are on the rise following the pandemic, studying how school-life balance affects the development of VET competences in online learning during COVID-19 can provide relevant information to enhance educational equity in the future. A longitudinal study was conducted employing an on-line questionnaire to meet the following aims: to explore the development of cross-disciplinary competences in VET during COVID-19; to identify different students’ profiles according to their school-life balance during the pandemic; and, to analyse whether the school-life balance was affecting competency development and propose improvements to training as a result. Results show that cross-disciplinary competences did not undergo important changes between the pre-pandemic scenario and during it, except for a decrease in metacognitive self-regulation. Similarly, most students reported having spent the same time studying before and during the pandemic. However, three main profiles of students are revealed, highlighting certain school-life imbalances. While all agree that remote teaching was critical in coping with the situation, differences were found by age and gender, with some participants experiencing more challenging situations. These results provide a fertile context for VET designers and teachers to generate new learning scenarios that meet all students’ potential needs.

**Supplementary Information:**

The online version contains supplementary material available at 10.1007/s12186-023-09314-1.

## School-life balance: factors and possible effects on VET students’ academic functioning before and during COVID-19

School-life balance involves integrating care, leisure time and workload in harmony with all dimensions of health and well-being (Gropel, & Kuhl, 2009). Thus, it has to do with how individuals—in this case, VET students—manage to participate in other activities and obligations connected with daily life (such as work, family, extracurricular activities, etc.) and at the same time balance them to fully engage with school demands. School-life balance is important for optimal academic functioning (Hatcher, & Hwang, 2020).

Melesk (2020) shows how women still spend more time on family responsibilities than men and how this results in barriers to their learning and academic development. In addition to gender, age can also affect school-life balance in VET: the older students are, the more likely they are to have domestic and care responsibilities at home. Spanish VET system has three levels: initial VET (*Formación Profesional -FP- inicial*), middle VET (*FP de grado medio*) and higher VET (*FP de grado superior*); this research focuses on middle and higher VET in the Spanish area of Catalonia. In Spain 49.28% of VET students (ISCED 3B and 5, the initial levels of Spanish vocational system) are over 21 years of age and 18.75% over 30, while 20 is the regular age for ending VET studies. If we analyse this distribution by gender (Table 1), women are more strongly represented in all age groups from age 25 and upwards, this figure reaching almost 50% more than men after the age of 35:

**Table 1:** Total VET enrolment distributed by age and gender 2019–2020

Age	Total	Men	Women
Under 18	25.08%	27.85%	21.85%
19	14.32%	15.03%	13.49%
20	11.32%	11.95%	10.59%
21	8.00%	8.57%	7.34%
22	5.91%	6.18%	5.59%
23	4.25%	4.35%	4.14%
24	3.22%	3.24%	3.20%
25	2.47%	2.38%	2.57%
26	2.05%	1.93%	2.19%
27	1.74%	1.57%	1.93%
28	1.52%	1.36%	1.71%
29	1.37%	1.19%	1.58%
30–34	5.77%	4.75%	6.97%
35–39	4.82%	3.77%	6.05%
+ 40	8.16%	5.88%	10.80%

Source: Based on figures from Educabase (2021)

All of the above are examples of characteristics that have a direct impact on the heterogeneity of student profiles. Much literature has been dedicated to analysing what are known as ‘non-traditional students’ in higher education, these being seen as atypical groups who have been more affected by external variables (work, responsibilities, family, etc.) (Sánchez-Gelabert, & Elias, 2017), but who, at the same time, have been found to exhibit greater resilience (Chung et al., 2017). According to this same literature, non-traditional students tend to drop out more often than their peers, even when performing well (Tieben, 2020). This may be influenced by the effect of age, due to increased family and work responsibilities, especially if access to studies has been delayed. Other interesting ideas revolve around contradictory results regarding the combination of studies and work. The number of weekly working hours, as well as poor conditions and stressful jobs, have also been identified as factors that determine these students' abilities to perform well (Metcalf, 2003).

During the pandemic, COVID-19 has affected lifestyles around the world and been proven to have an impact on domestic relationships and patterns of care, either generating or increasing imbalances in school-life balance, especially for those who were already vulnerable (Babb et al., 2022). From a social justice perspective, Dietrich et al. (2021) have shown how the closure of schools has aggravated social inequalities, with different results according to social background and learning environments. For instance, time and financial pressures have been particularly acute when added to caregiving responsibilities, especially for those with children living at home (Collins et al., 2021). Those students who are simultaneously juggling studies with caregiving and work demands have found themselves in a very difficult position, resulting, at the very least, in psychological and emotional stress (Doolan et al., 2021; Maulana, 2021). We know very little about how students have been coping with these tasks – which are sometimes seen as incompatible – or how this has affected their academic functioning.

### Cross-disciplinary competence development in VET during COVID-19

As VET entails practice-oriented teaching, greater difficulties have been found when adapting learning to digital environments. This practice-oriented learning can lead to “distance learning” being perceived as having less validity in vocational than in general upper-secondary education; in fact, remedial activities have been implemented to assess learning gaps generated by the limitations of distance learning (OECD, 2021). Despite this, however, Görl-Rottstädt et al. (2022) show how the mass introduction of virtual scenarios in VET has an important impact on teaching and learning processes and affects the development of students’ cross-disciplinary competences.

According to some studies (Choi et al., 2020; García-Alberti et al., 2021), students have encountered difficulties in developing competences to become autonomous and active learners during the pandemic. Specifically, there have been cases of students spending less time studying, showing less commitment and motivation, and a reduction in self-confidence and security (García-Alberti et al., 2021), especially regarding their level of preparation to enter the labour market and professional practice (Choi et al., 2020). In relation to this, cross-disciplinary competences have been highlighted as the area where most difficulties are found. The concept of cross-disciplinary competences refers to a broad set of knowledge, skills, and work habits believed to be critically important to success in in today’s world; it relates to the concept of twenty-first century skills and is widely used in reference to interdisciplinary, transversal and transferable competences (Glossary of Education Reform, 2014).

Work on communication skills has been particularly affected and thus identified as an area for improvement in most of the aforementioned studies. In training courses that focus on human relations, such as those related to health, barriers were found when establishing empathic conversations due to the lack of non-verbal communication on distance learning courses (Ludwig et al., 2021). This lack of or decrease in non-verbal communication also generated the perception of “artificiality” in some students and learning moments (Ullmann-Moskovits et al., 2021). In the same line, Hartmann, Kaden and Strohmer (2021, p. 2) found that emotional intensity decreased in group dynamics and discussion “compared to face-to-face sessions, which limited the authenticity and quality of the learning experience”.

The lack of physical contact with others during the pandemic is also expected to have had an effect on teamwork, which goes hand-in-hand with human interaction. It can be assumed that learning in a virtual environment, where there is far less interaction with others than in a face-to-face situation, will have decreased teamwork skills development among students (Brown, Te Riele, Shelley, & Woodroffe, 2020).

Furthermore, metacognition has been considered essential due to its positive correlation with online learning performance and effective educational strategies (Anthonysamy, 2021). One of the consequences of educational centres closing their doors is that students have much more responsibility for their learning, making metacognitive awareness and regulation more relevant for educational success. In this sense, Sukarno and El Widdah (2020) have shown how a high metacognition score has correlated with better self-regulated study during the COVID-19 pandemic, while Öztürk (2021) has illustrated how students who received metacognition-based education have displayed better learning in the cognitive fields.

In this context, our study evaluates the development by VET students of the mentioned cross-disciplinary competences, which have been more affected by pandemics, according to school-life balance profiles. On the one hand, we believe some students may have been more negatively affected than others by the pandemic in general and, specifically, by the transition to virtual learning. And on the other, given that so-called non-traditional students have been found to be more resilient in higher education, we believe that in VET they may have had more resources to overcome or cope with the situation.

Following these insights, this article has three aims: (a) to explore VET students’ development of cross-disciplinary competences during the COVID-19 pandemic; (b) to identify different student profiles according to their school-life balance during the same period; and (c), to analyse whether school-life balance affected the development of cross-disciplinary competences in VET during the pandemic and propose adaptations and improvements to training as a result.

## Method

### Design

A longitudinal study (three waves of data collection) was designed with a questionnaire within a broader research project. However, for the purposes of this article, only the results from the first two waves (W1 and W2) of data collection are considered. These were gathered in October 2019 and January 2021, corresponding to two key moments: VET students’ first academic year, and the initial stages of their apprenticeship. Despite the limitations of considering only two data points, the decision of basing the analyses on these two waves was justified by the opportunity to isolate the timeframe where the pandemic affected social and educational processes the most. In Spain, the lockdown occurred between March 2020 and June 2020; from September 2020, a hybrid teaching modality was developed; around January 2021, regular face-to-face education was progressively adopted again.

### Instruments and measures

The research tool used for the first wave (W1) was an ad hoc questionnaire in Catalan, which was based on various validated scales. It included a first part with questions requesting demographic and academic information, mainly multiple-choice items (age, gender, academic pathways, work experience and main reason for attending the VET programme). The second part of the instrument was composed of questions regarding cross-disciplinary competences, as well as variables related to motivational and SRL strategies. Finally, students had to self-evaluate their accomplishment of five key technical skills specifically related to their VET programme.

In W2, the same scales were applied, together with some questions enquiring about the effects of school-life balance during the pandemic. Students were asked to report whether they had children, and of what ages, and whether they had other people in their care (children and/or ill, elderly and dependent people). For all of these situations, they were specifically asked how much time their family responsibilities took up prior to and during the pandemic; how much time they dedicated to their studies prior to and during the pandemic; and which other time-consuming activities they had been engaged in since W1, such as paid work, voluntary work, internships, etc. Finally, in an open-ended question they were asked to explain which factors had impacted their studies since the outbreak of the pandemic.

Among the cross-disciplinary competences, three different summative scales which measured communication, teamwork and metacognitive self-regulation will be here presented. Exploratory factor analysis was used to gather evidence of construct validity, using data from the W1 (the same analyses were later performed with data from the W2 to check that the structure had not changed). Maximum likelihood was used as an extraction method, and factors were rotated using the direct oblimin method (delta = 0). To determine the number of factors, Kaiser’s K1 criterion was used, together with inspection of the scree plot; the interpretability of the factors was also considered.

The *communication* scale was designed ad hoc following Duran (1992) and for the purpose of framing the acquisition of communicative competence in this educational stage. After the first 14 items were drafted, the scale was subjected to field validation using a sample of 39 students. As a result, a further four items were added, and others were modified to facilitate their understanding. After applying these to 509 VET students in a pilot application of the questionnaire, the scale was refined to its final version of nine frequency-type items, ranging from 1 (Never) to 5 (Always). The analysis showed two factors that explain 59.1% of the variance. The first factor included four items related to emotions (feeling nervous, having insecurities when speaking in public or with other people, etc.); α_w1_ = 0.812; α_w2_ = 0.836, while the second included five items on communication effectiveness (strategies to make explanations clearer, voice modulation, etc.); α _w1_ = 0.718. α _w2_ = 0.721.

The *teamwork scale* (Lower, Newman & Anderson-Butcher, 2015) was translated into Catalan and then back-translated into English with the help of two native speakers fluent in both English and Catalan. The final version was then compiled by the researchers. Initially, the ten items were used, but three factors emerged from the factorial analysis, one of which had only two items (1 and 2). The aforementioned authors had already warned that these two items were problematic. They were therefore eliminated, and the EFA yielded a solution of two factors capable of explaining 57.6% of the variance. The first (Items 3, 9 and 10) included components of the competence related to individuals’ confidence in their abilities to lead, communicate and work in a team; α_w1_ = 0.714; α_w2_ = 0.737. The second (Items 4, 5, 6, 7 and 8) included the behavioural component of the competence; α_w1_ = 0.761; α_w2_ = 0.733.

Finally, the *metacognitive self-regulation* scale followed the aforementioned procedure to translate it into Catalan from Martínez and Galán’s (2000) Spanish version of Pintrich’s (1991) Motivated Strategies for Learning Questionnaire (MSLQ). The scale comprised 12 items from the metacognitive self-regulation factor on Pintrich’s original scale: the first (number 33 on the original version) and eighth (corresponding to number 57) items were removed. The analysis carried out using the pilot sample to test the scale yielded a unique factor that explains 39.41% of the variance; α_w1_ = 0.817; α_w2_ = 0.829 (this was higher than the mean alpha of 0.754, according to the meta-analysis by Holland et al., 2018).

All of the items used for these analyses and the dataset are provided as supplementary material.

### Participants and research setting

The participants were students enrolled in VET at different schools who were invited to participate in the study following purposive sampling. According to the main goals of the broader project, ten VET programmes were initially selected from the initial Spanish VET system (ISCED 3B and 5), which comprises two sectors: healthcare and technology. These two sectors were previously identified, by means of interviews to experts, as the most demanded ones by the Catalan productive system of the future (2030).

All schools in Catalonia that offered these ten programmes (face-to-face and in both dual and non-dual mode) were contacted by the research team during the academic year 2019–2020. Of the 73 schools identified, 45 agreed to participate; which resulted in the inclusion of eight VET programmes in the sample (Table 2), since two of the programmes were offered by schools who did not consent to participate. The research sample for the first wave (W1) ultimately comprised all first-year students from the 45 participating schools who had given their informed consent (*n* = 1,618).

**Table 2:** Distribution of participants by VET programmes

ISCED level	VET programme	% of sample
5	Networked Computer Systems Administration	22.6
3	Care for Dependent People	26.01
3	Care nursing assistants	3.03
5	Multiplatform Application Development	9.97
5	Web Application Development	5.05
3	Electrical and Automatic Installations	7.2
5	Social integration	21.21
3	Microcomputer Systems and Networks	4.92

Of the 1,618 respondents in W1, 753 also responded to W2 (46.5%). The pandemic played a role in lowering the response rate, since not all groups were attending face-to-face classes at the time of W2 due to quarantine. In the present article, results are only reported for those students who responded to both questionnaires.

To verify that the data loss in W2 did not follow a pattern, the basic profile data of the participants who responded to both waves were compared, as well as the missing values in W2. The analysis reported that the mean age was the same for both groups and the chi-square tests did not reveal significant associations in the distribution by gender (*χ*^*2*^ = 8.763, df = 4, *p* = 0.086), work experience (*χ*^*2*^ = 2.252, df = 2, *p* = 0.324), or by the fact of having responded or not to the questionnaire in W2.

Overall, out of the 753 who responded to the questionnaires in both waves, 47% were girls, 52.2% boys, and 0.8% preferred not to express their gender. The mean age was 20.52 (SD = 5.54). Approximately two thirds (63.9%) had work experience, and in 47.7% of cases this was related to their current vocational studies. The main reasons they gave to enrol on the course were as follows: 27.4% chose their VET programme due to personal interest or vocation; 26.4% to access a higher qualification; 18.7% to find a job in that sector; and 13.8% to advance their career opportunities and development. While 98% of the students surveyed did not have children, 28.7% reported having taken care of others (children, the elderly, siblings, cousins, sick family members, etc.) during the pandemic.

### Data collection

For on-line administration of the questionnaire, it was agreed with course coordinators and tutors that the latter would be responsible for providing students with the access link, monitoring the process, and ensuring its correct completion. Online administration mainly took place in class on the days agreed between the research team and the schools, and typically took 15 min. Also, participants were informed of their rights and signed a consent form, which also allowed them to refuse participation or to withdraw at any time. The Research Ethics Committee [details removed for peer-review] had previously approved the study design and implementation, including all consent procedures. Data confidentiality was guaranteed, and participants were at least 16 years old and had full capacity to decide regarding completion of the survey.

### Data analysis

Analyses were performed using SPSS v.25, and databases were matched using the IDs reported by students. A two-step cluster analysis was performed using the Log-Likelihood Distance to reveal natural groupings in the dataset with similar characteristics. In so doing, four variables were included in the analysis: gender, having children, having taken care of other people and age. After some preliminary analyses, a fixed number of three clusters was applied in order to facilitate the following analyses. Using the following open-ended question “How has the pandemic affected you?”, we also undertook a content analysis to identify, classify and tabulate differences and similarities among the identified clusters. The process of analysing the content of responses produced a number of inductive themes (i.e. lack of socialization; mental health issues; lack of motivation; work, personal and/or academic overload; etc.), which were used to characterize the clusters.

We also conducted simple and mixed (split-plot design) repeated-measures analysis of variance (ANOVA) to assess the statistical significance of changes in participants’ development of competences over time and across groups. Assumption of normality was verified by visually inspecting the histograms, Q-Q and detrended Q-Q graphs. Some deviations from normality were observed, especially negative asymmetries; however, the ANOVA is fairly robust to these deviations, as long as the other assumptions are met.

As for homoscedasticity, this was verified using Levene’s test. It was fulfilled in all cases (*p* > 0.05), except for the two factors of communicative competence. In these cases, Games-Howell post-hoc tests were used, as opposed to Tukey's tests, which were used for post-hoc comparisons if the assumption was fulfilled. The Bonferroni correction was used for all pair-wise comparisons. The assumption of compound symmetry was verified using Box's test of Equality of Covariance Matrices, which resulted in *p* > 0.05 in all cases except for metacognitive self-regulation. In this case, Pillai's trace criterion was used, a robust statistic to counter departures from the multivariate homogeneity of variance–covariance matrices (Tabachnick, Fidell & Ullman 2007). The assumption of subject-treatment independence was verified using Tukey’s non-additivity test. In all cases, the analyses returned *p* values > 0.05, which suggests the independence assumption was fulfilled.

## Results

The results are presented in accordance with the aims outlined for this article.

### Development of cross-disciplinary competences before and during*** COVID-19***

In order to address the first aim, repeated measures of ANOVA modelling were performed. The analysis of cross-disciplinary competences between the first and second waves—i.e. before and during the pandemic—revealed no significant differences, except in the cases of communication (the efficacy component) and metacognitive self-regulation. Even so, these differences were very small considering the partial eta-squared value (all relevant outputs are presented in Table 3). In the former case, perceived competence seemed to increase, whereas with metacognitive self-regulation participants reported lower perceived levels in their second academic year (W2).

**Table 3:** Repeated ANOVA measures (cross-disciplinary competences over time)

Variables	ANOVA results	Mean (SD)
Communication (Emotional component)	*F*_(1. 745)_ = .017* p* = .896	W1: 3.78 (0.88) W2: 3.78 (0.90)
* Communication (Efficacy component)	*F*_(1. 745)_ = .029* p* = .028 *η*^*2*^_*p*_ = .006	W1: 3.23 (0.73) W2: 3.29 (0.71)
Teamwork (Self-confidence component)	*F*_(1. 731)_ = 1.604 *p* = .206	W1: 3.77 (0.83) W2: 3.81 (0.82)
Teamwork (Behavioural component)	*F*_(1. 731)_ = 0.000 *p* = .983	W1: 4.09 (0.67) W2: 4.09 (0.65)
* Metacognitive self-regulation	*F*_(1. 731)_ = 6.493 *p* = .011 *η*^*2*^_*p*_ = .009	W1: 3.36 (0.70) W2: 3.29 (0.71)

Note: * *p* < .005

### Students’ school-life balance profiles during the pandemic

Regarding the second aim, using a two-step cluster with the 753 respondents, three student profiles were identified with respect to school-life balance differences during COVID-19. The three clusters deriving from the algorithm had a good quality index (the silhouette measure of cohesion and separation being 0.75), with four variables, in the following order of importance: having taken care of other people (importance: 1), gender (importance: 0.8), having children (importance: 0.06) and age (importance: 0.03).

As a result, the group pertaining to cluster 1 (*n* = 325; 44%) was characterized by men without children who were mostly young (mean age 20.4; maximum 46) and who had not taken care of others during the pandemic. The content analysis of the open-ended responses revealed that the main factors to have impacted students’ learning during the pandemic were remote teaching (both online mode and the perceived lack of organization and communication from the school), followed by lockdown. Regarding this aspect, the participants reported that they were highly affected by not being able to socialize, which in turn impacted their mental health and motivation.

The respondents comprising cluster 2 (*n* = 210; 28.5%) were mainly women (69%) with children (4.14%) or who have assumed responsibility and cared for others during this period (95.7%); this cluster also included the oldest respondents in the sample (mean age 21.9; maximum 57). We can characterize this cluster as non-traditional students. The aspect that emerged as most relevant from the qualitative analysis was related to mental health issues, stress and lack of motivation. Some participants reported a deterioration of pre-existing conditions, others panic attacks and anxiety, which were ascribed to academic, work and personal overload, to the pandemic itself, and to the lockdown. Other factors that were mentioned as impacting their learning were related to remote teaching, similarly to cluster 1.

Cluster 3 (*n* = 203; 27.5%), on the other hand, was characterized by women without children who—similarly to the first profile—were younger (mean age 19.3; maximum 39) and had not taken care of other people or assumed similar responsibilities. The qualitative analysis revealed a somewhat different situation from the other two clusters: although the first aspect that seems to have impacted on their learning was reported as mental health issues, from the participants’ responses, these were less related to the lockdown and more to the social and healthcare situation, as well as uncertainty. One word that emerged in this cluster but not among participants in the other clusters was “anguish”. These participants also more frequently mentioned personal and family problems, such as problematic relationships and the loss of loved ones.

In order to describe these three groups, Table 4 presents how much time they reported dedicating to study. It is apparent that the participants in cluster 1 (men without care responsibilities) expressed that they already spent less time on this than the other two groups prior to the pandemic, with 40.9% of them spending only some days per week studying. The same pattern seems to be present after the onset of the pandemic; however, it is also worth noting that a higher percentage of participants in cluster 3 (36.1%) reported that they did not study every day of the week.

**Table 4:** Reported time dedicated to study before and after the outbreak of the pandemic by cluster

		**Cluster 1**	**Cluster 2**	**Cluster 3**
**Time dedicated to study before the pandemic**	More than 7 h per day	1.2%	4.8%	2.5%
	5–7 h per day	5.8%	9.5%	7.9%
	2–4 h per day	25.2%	30.5%	30.7%
	Less than 2 h per day	26.8%	28.6%	31.2%
	Some days per week	40.9%	26.7%	27.7%
**Time dedicated to study after the pandemic**	More than 7 h per day	2.5%	4.8%	1.0%
	5–7 h per day	8.6%	14.3%	9.9%
	2–4 h per day	23.7%	26.7%	30.7%
	Less than 2 h per day	23.4%	25.2%	22.3%
	Some days per week	41.8%	29.0%	36.1%

It is worth mentioning here that the pandemic had a different impact on the amount of time each group had available to study: as Table 5 shows, there was no change at all for more than half of the participants in each group, while both a negative (less time available) and a positive change (more time available) were experienced by participants of all three groups. The highest percentage of respondents reporting a negative change was found in cluster 3: younger women who do not have children and did not take care of others during the pandemic.

**Table 5:** Change in time dedicated to studying and taking care of others, before and during the pandemic

		**Cluster 1**	**Cluster 2**	**Cluster 3**	**Cluster 1**	**Cluster 2**	**Cluster 3**
		**Freq**	**%**	**Freq**	**Freq**	**%**	**Freq**
Study time	Less time dedicated to study	54	16.6%	43	20.5%	52	25.7%
	No change	195	60%	123	58.6%	112	55.4%
	More time dedicated to study	76	23.4%	44	21%	38	18.8%
Care responsibilities	Less time dedicated to care duties	0	0%	34	16.9%	1	25%
	No change	0	0%	99	49.3%	2	50%
	More time dedicated to care	1	100%	68	33.8%	1	25%

As for the impact of the healthcare situation on time dedicated to taking care of others, this was only observed in cluster 2 (since only participants who had these responsibilities had to respond to this section of the questionnaire). Of these, nearly half experienced no change, while 33.8% reported that they had spent more time taking care of other people since the outbreak of the pandemic.

### How school-life balance during the pandemic affected the development of cross-disciplinary competences

Having identified the above profiles, mixed ANOVAs were performed in order to ascertain whether the development of cross-disciplinary competences differed among the three groups with different school-life balance situations.

With regard to communication competence, a significant main effect of the cluster (F_(2, 728)_ = 3.910, *p* = 0.020) was found in the emotional component. This result suggests that there are differences between at least two of the clusters in terms of the development of cross-disciplinary competences. However, the interaction of the between-subjects effect (belonging to one of the clusters) and the within-subjects effect (the academic year) was not found to be significant, indicating that the variation in competence development between people with different school-life balance situations followed the same pattern (with very few changes), even if the starting point differed with regard to level of competence.

To identify where these differences were, post-hoc test comparisons were performed (the Games-Howell test, and, since the homoscedasticity assumption was not met in this case, Levene’s test *p* < 0.05). The Bonferroni correction was applied. These analyses revealed significant differences between clusters 1 and 3 (*p*_*adj*_ = 0.031), with a higher level of perceived competence in cluster 1 (the difference being 0.191). The other pair-wise comparisons did not display statistically significant differences (*p*_*adj*_ > 0.05).

As for the efficacy component of communication competence, the interaction between the effect of the cluster and the year did not prove significant either (*p* > 0.05). However, a significant main cluster effect was found (*F*_(2, 728)_ = 3.219, *p* = 0.041). Games-Howell post-hoc comparisons showed a significant difference (*p*_*adj*_ = 0.044) between clusters 2 and 3, with a higher level of perceived competence in cluster 2 (the mean difference being 0.16). That is to say, those students who have taken care of others during the pandemic reported higher scores in the efficacy component of communication competence.

Table 6 shows descriptive data (mean and statistic deviation) for all groups and both components of communication competence.

**Table 6:** Mean and statistical deviation for communication competence, by cluster and year (W1 and W2)

Cluster	Emotional component		Efficacy component	
	**W1**	**W2**	**W1**	**W2**
1 (men, traditional students)	3.82 (0.87) ↑	3.86 (0.86)	3.23 (0.70)	3.31 (0.68)
2 (non-traditional students)	3.81 (0.84)	3.81 (0.84)	3.32 (0.76) ↑	3.36 (0.68)
3 (women, traditional students)	3.70 (0.88) ↓	3.78 (0.90)	3.16 (0.78) ↓	3.19 (0.76)

Note. The boxes marked in grey indicate where a significant difference (*p*_*adj*_ < .05) was found between clusters. No significant within-subject effect nor interactions were found

↑ Higher values

↓ Lower values

Regarding teamwork competence, in the self-confidence component, only the between-subjects effect was found to be significant (F_(2, 723)_ = 4,381, *p* = 0.013), indicating no apparent evolution in competence level. Although this stability was reproduced across the three clusters, one difference was generated by the school-life balance situation. Tukey’s post-hoc comparison suggested that the significant difference (*p*_*adj*_ = 0.015) was between clusters 1 and 2, with a higher perceived competence in the latter (the difference being 0.18).

As for the behavioural component of teamwork competence, again no within-subjects nor interaction effects were found to be significant to explain the variance among the scores. That being said, the clusters did have a significant main effect (F_(2, 723)_ = 14,171, *p* < 0.001). Post-hoc Tukey comparisons indicated that the scores in cluster 1 were significantly lower (*p* < 0.001) than those in clusters 2 and 3 (the difference being 0.22 in both cases).

Table 7 presents descriptive data for all groups for both components of teamwork competence.

**Table 7:** Mean and statistical deviation for teamwork competence by cluster and year (W1 and W2)

Cluster	Self-confidence component		Behavioural component	
	**W1**	**W2**	**W1**	**W2**
1 (men, traditional students)	3.69 (0.85) ↓	3.71 (0.85)	3.96 (0.69) ↓	3.98 (0.63)
2 (non-traditional students)	3.87 (0.80) ↑	3.90 (0.80)	4.20 (0.65) ↑	4.17 (0.65)
3 (women, traditional students)	3.80 (0.81)	3.86 (0.82)	4.20 (0.62) ↑	4.18 (0.64)

Note. The boxes marked in grey indicate where a significant difference (*p*_*adj*_ < .05) was found between clusters. No significant within-subject effect nor interactions were found

↑ Higher values

↓ Lower values

Finally, with regard to metacognitive self-regulation, the results of the ANOVA suggested that both principal effects were significant: academic year (F_(1, 723)_ = 7,603, *p* = 0.003), there being a slight decrease in the second year; and belonging to a specific cluster (F_(2, 723)_ = 10,725, *p* < 0.001). However, the interaction did not appear to be significant (*p* > 0.05), indicating that perception of this competence differed across the three work-life balance situations. That being said, regardless of the cluster, perception of metacognitive self-regulation decreased equally from the first to the second year (Table 8).

Post-hoc Tukey comparisons revealed significant differences between clusters 1 and 2 (*p* < 0.001), with cluster 2 presenting higher scores (the mean difference being 0.23); and between clusters 1 and 3 (*p* = 0.002), with participants in cluster 3 reporting higher values (the mean difference being 0.18).

**Table 8:** Mean and statistical deviation for metacognitive self-regulation by cluster and year (W1 and W2)

Cluster	W1	W2
1 (men, traditional students)	3.22 (0.69) ↓	3.19 (0.67)
2 (non-traditional students)	3.48 (0.68) ↑	3.40 (0.72)
3 (women, traditional students)	3.45 (0.71) ↑	3.33 (0.75)

Note. The boxes marked in grey indicate where a significant difference (*p*_*adj*_ < .05) was found between clusters. Also, a significant within-subjects effect was found. Interaction was not significant

↑ Higher values

↓ Lower values

## Discussion and conclusions

The pandemic may have intensified social inequalities and destabilized the school-life balance, resulting in worse cross-disciplinary competence development among ‘non-traditional’ students with caring responsibilities. In this research, we have explored how the situation has affected VET, and found three main profiles of student. The largest cluster comprised young men with no caring responsibilities; the second were mainly women who cared for relatives—this group also had a higher mean age than the other groups and can be considered non-traditional students; and the third comprised of young women with no caring responsibilities. The qualitative analysis detected differences between the groups regarding factors impacting learning during COVID-19. Although all three groups highlighted remote teaching as one of the most critical factors, mentions of mental health issues, stress and lack of motivation were significantly higher among the second and third groups – mainly women – than the first – all men. These gender differences were also intersected by differences in age: family care and work were found to be major determining factors among members of the older group, while the younger group highlighted uncertainty and anguish regarding the social and healthcare situation.

With regards to time dedicated to studying, our results reveal that most of the sample of VET students spent almost the same amount prior to and during the pandemic; however, the group of young women with no caring responsibilities reported dedicating less time to study during the pandemic. This finding partially aligns with that reported by Grätz and Lipps (2021), who found a large drop in hours spent studying during the pandemic in secondary and university education, with an average decrease from 35 to 23 h per week. This loss was strongly mediated by age, with younger students reducing their study time considerably more than older ones. In our study, the younger cluster of women VET students followed a similar pattern, this being the group with the largest decrease in time spent studying. This reveals a strong mediating role of gender on time spent studying, which it was not possible to analyse in the Grätz and Lipps study (2021). Our results complete their findings, since together they analyse the effects of the COVID-19 pandemic on different educational paths for young people: secondary education, VET and university.

Interestingly, school-life balance was not found to clearly impact on the group of students with caring responsibilities in terms of time dedicated to studying.

When it comes to learning outcomes, no important changes were identified with regard to competences between the pre-pandemic and pandemic scenarios, except for a perceived decrease in metacognitive self-regulation. One possible partial explanation for this might be the Dunning Kruger effect (Dunning, 2011): people who have large deficits in certain abilities are also not able to acknowledge their deficiencies. Paradoxically, as they develop their skills, their perception will be of having fewer skills as they are now able to detect the deficits. In addition, competences are very slow to evolve, and their full development happens in real contexts of practice. All that being said, our findings for VET follow the trend described by other studies in different educational contexts, whereby a decrease is detected in capabilities such as active learning, autonomy, commitment and motivation, all closely related to metacognitive competence. In one study on higher education students, García-Alberti et al. (2021) found a decrease in motivation and engagement and a lower performance among those students who were already low-profile prior to lockdown. This demotivating effect also affects final-year university students in their transition to work by impacting their sense of confidence and preparedness (Choi et al., 2020). Similar effects were reported by González-Calvo et al. (2020) for students who were unable to complete their work experience due to the pandemic, these reporting insecurities and low confidence in their preparation to become professionals.

If we analyse these groups in greater depth, we find that, surprisingly, the cluster characterized by family responsibilities and elder students (mainly women) did not suffer the foreseeable decrease in competences that might occur as a result of barriers to learning caused by caring for children. In fact, it has been shown that women suffered a “double working day” (Farré & González, 2021) during the pandemic in order to maintain their work-life balance. However, in our study of VET students, those with caring responsibilities were found to have a higher competence level than the other students, despite the unbalanced situation. Caring responsibilities seem to fall mainly on women; thus, it is reasonable to think that this group of students, who already had an established routine balancing personal and academic life prior to the pandemic, did not experience much of an impact. These findings also reaffirm the higher resilience of non-traditional students found by Chung et al. (2017).

Despite the little change in levels of competence between the waves, significant differences were found between clusters regarding competence level starting point. While the group of young men scored significantly higher than the young women’s group on communication competences, they also scored significantly lower on teamwork and metacognition. These significant gaps between the two groups of younger students could be related to how stereotypes shape intellectual identity and performance regarding gender in VET, resulting in inequalities during and after educational training (Niemeyer & Colley 2015). In this respect, Steele (1997) argued that women are more likely to develop gender-stereotyped patterns in order to fit males’ views. This may partially explain the lower communication development among young women, since they may have less frequent and lower quality interaction with teachers in terms of feedback or questioning (Sadker, 2002). With regard to teamwork competence, Hartman and Hartman (2006) found a much more positive attitude towards teamwork among women than men. Takeda and Homberg (2014) presented similar results, reporting lower performance among all-male groups and less collaborative behaviours by solo males in female groups. This would suggest that gender stereotypes remain a problem in VET at the very start of the training process and, therefore, orientation pathways still have room for improvement.

These findings provide insight into how learning in VET has been affected by COVID-19, as well as the influence of school-life balance, and open up new scenarios for future research and decision-making by policymakers. Firstly, they show the importance of good professional guidance in VET, and strong motivation, which are often connected; a lack of these may be the underlying cause behind the worse performance of students – the youngest – with fewer family duties. As Yuk-Kwan et al. (2021) have pointed out, older students are mature learners, experienced, self-motivated, self-directed and outcome-driven. The fact that the older students are, the more involved they are with their duties demonstrates how important it is to understand VET not as the only remaining option for youth discarded from university, but as a valid pathway for motivated students with a solid professional orientation. It also shows how necessary it is to change the way students and teachers understand VET.

Some of the limitations are related to the sample used. On the one hand, STEM educational options are characterized by a large concentration of man, while healthcare options are much more represented by woman. So, in future studies, it may be interesting to analyse the effect of the pandemic on competence development taking into count the differences between course composition in terms of gender and age. Also, the intensity of the pandemic itself posed limitations; the speed, instability and complexity of the situation limited the possibility of studying the phenomenon by including other objective tests or sources apart from the self-perception questionnaire. On the other hand, the conditions of the pandemic made it difficult to obtain a greater number of responses. Another interesting line for further studies may be to analyse the difference in competence development in medium and high-level VET (ISCED 3B and 5), considering gender, age and time available to study as key variables.

Since the onset of the pandemic, online learning has been more present in VET and education in general, but there is still work to be done. The results show the importance of reorganizing the VET methodology under an e-learning base to guarantee the acquisition of professional competences. In this sense, it is also necessary to analyse and improve the online teaching of cross-disciplinary competences for VET centres that want to guarantee a global online education experience. Furthermore, from a social perspective, older students with family responsibilities, especially women, comprise an important group in VET, and they have been found to be more resilient than other students; this fact shows the need for institutions to recognize this situation which is more frequent in women. This situation opens up a new opportunity for VET designers and teachers to address all students’ needs and thereby enhance educational equity in the future (Majumdar & Araiztegui, 2020). If, in the words of Avis et al. (2020), the pandemic has led to a re-conceptualization of VET that enhances its emancipatory possibilities, then it is vital to respond to the needs of older students, especially women. These findings may therefore prove useful for policymakers in developing a new VET scenario.

## Supplementary Information

**Additional file 1.**

## Data Availability

The data that support the findings of this study are openly available in figshare at 10.6084/m9.figshare.22117703.v3.
